# Determinants of student’s physical activity: a 12-month follow-up study in Ningxia province

**DOI:** 10.1186/s12889-021-10525-1

**Published:** 2021-03-16

**Authors:** Wei Huang, Xiangrong Shi, Yujie Wang, Xiaoling Li, Pengpeng Gao, Jieguo Lu, Jie Zhuang

**Affiliations:** 1 School of Kinesiology, Shanghai University of Sport, 399 Chang Hai Road, Shanghai, 200438 China; 2 Department of Pharmacology & Neuroscience, University of North Texas Halth Science Center, Fort Worth, TX USA; 3 School of Physical Education, Ningxia University, 489 He Lan Shan West Road, Yinchuan, 750021 Ningxia Province China; 4 School of Physical Education, North Minzu University, 204 Wen Chang North Road, Yinchuan, 750021 Ningxia Province China; 5 Physical Education, Health and Art Office, Ningxia Provincial Department of Education, 127 Shang Hai West Road, Yinchuan, 750004 Ningxia Province China

**Keywords:** Physical activity, Children, Adolescent, School environment, Neighbourhood environment

## Abstract

**Background:**

Physical activity has many health benefits for children and adolescents. However, the prevalence of physical inactivity in school-aged children and adolescents remains high in China. Many factors impact the levels of moderate and vigorous physical activity (MVPA) among students. This study investigated the factors associated with students’ MVPA levels and the determinants of changes in their MVPA behaviour.

**Methods:**

This is a longitudinal study with a 12-month follow-up. The study samples were obtained from 2016 and 2017 Physical Activity and Fitness in China—The Youth Study, and they included 1597 students (aged 9–18 years) from 31 primary, junior high, and high schools in Ningxia Province. Factors related to the individual (Age, Sex, BMI and attitude to PA), school (school exercise facility, PE class, teacher support, PA time and PA environment) and neighbourhood (free skill training, sport events, sport organization, sport facility) factors were measured via questionnaire at baseline and after 12 months. Multiple logistic regression was performed to examine the factors that impact students’ MVPA level and the determinants of changes in students’ MVPA behaviour.

**Results:**

There was no difference in students’ MVPA levels between 2016 and 2017. Boys were more physically active than girls at baseline (RR 1.55, 95% CI 1.10, 2.20). Neighbourhood factors associated students’ MVPA levels were significant, but all of these factors (neighbourhood sport events, organizations, facilities, etc.) were removed from the longitudinal model. Individual and school factors were important for students’ MVPA maintenance and positive development (e.g., gender, attitude, school PE class and PA time).

**Conclusions:**

In conclusion, both neighbourhood and school factors may affect students’ MVPA, but school appears to plays a more critical role in maintaining and promoting students’ MVPA levels. In addition, individual factors may be more important than school and neighbourhood factors in influencing students’ MVPA levels. Our research demonstrates that students’ attitudes towards PA and school factors should be considered targets for future intervention programmes to promote MVPA. More education programmes may help enhance students’ attitudes towards PA, but more studies with large samples and objective assessments are needed to explore the determinants of MVPA.

**Supplementary Information:**

The online version contains supplementary material available at 10.1186/s12889-021-10525-1.

## Background

Cumulative evidence has demonstrated that physical activity (PA) is beneficial for the physical and mental health of children and adolescents, such as reducing the prevalence of overweight/obesity, cardiovascular disease, type 2 diabetes and even mental health [1, 2]. In contrast, the disadvantage of sedentary behaviour (SED) has been identified [3–5]. Childhood physical activity (PA) behaviour can be traced to adulthood [6], indicating that PA-related health benefits in adulthood may derive from an active lifestyle early in the lifespan. Although the benefit of PA is evident, approximately 80% of adolescents globally (those 13–15 years old) do not meet the recommended guidelines [7]. As early as 2010, the World Health Organization recommended that children and adolescents aged 5–17 years need at least 60 min of MVPA daily, but only 22.7% of students met the recommendation in China [8]. By 2016, approximately 70% of Chinese students had not met the PA recommendation [9]. A national health policy named “Healthy China 2030” was issued in 2016 to promote healthy lifestyles and physical fitness, recommending that school-aged children participate in physical activity for 1 h daily to achieve the goal of a 25% “excellent” ratio of physical fitness assessment. Even so, only 34.1% of students met the recommendation of 60 min of MVPA [10].

Both school and neighbourhood are important for students’ physical activity participation [11], and many studies have explored the factors associated with MVPA to develop an intervention to encourage such activity. For example, the school environment plays a crucial role in students’ engagement in PA [12, 13]. The PA-friendly school environment is positively associated with students’ PA participation [14]. Meanwhile, the extramural activity also effectively promotes MVPA, which provides more opportunities for students to participate in PA, such as neighbourhood-based PA facilities and organizations [15–18]. Moreover, students’ PA behaviour is also influenced by others when they are in school and the neighbourhood, including others’ support for PA or behaviours. A systematic review revealed that students with physically active parents are more likely to have an active lifestyle [19], as well as peer support [20, 21]. Although many studies have investigated the correlates of PA among children and adolescents, these studies have not contributed to preventing the decline in PA among children and adolescents [22]. Therefore, it is important to investigate the determinants of changes in PA behaviour, which will help us to determine the factors that increase PA levels and will be targets for intervention. One study reviewed the determinants of change in physical activity in children and adolescents, but few of the variables studied were consistently associated with changes in physical activity [23]. More research is needed to investigate the factors that influence the change in PA in children and adolescents. Recently, a longitudinal study based on the social-ecological model of healthy behaviour found that child-reported parent encouragement and social spaces contributed to positive changes in students’ PA behaviour and that factors from different domains were associated with students’ PA behaviour [24]. However, they did not investigate the impact of friend-related factors and factors for sustaining students’ PA level, which can be regarded as factors that prevent the decline in PA with age. Therefore, more research is needed to investigate factors influencing changes in PA among children and adolescents at multiple levels and to identify the potential factors that sustain or increase PA.

In the current study, we investigated the associations of students’ MVPA levels with individual and school and neighbourhood environment factors at a 12-month follow-up. The outcomes of this investigation could aid in developing effective interventions to promote awareness of PA and increase MVPA levels for children and adolescents and identify guidance for taking the next step in health policy-making for students.

## Methods

### Study design

This was a longitudinal survey study. The baseline sample was taken from 2016 Physical Activity and Fitness in China—the Youth Study (PAFCTYS) project, a nationwide survey of PA and fitness among Chinese school-aged children and adolescents. Thirty-one primary, junior-high and high schools were randomly selected from 8 counties/cities at Ningxia province in China. The schools were evenly distributed in urban and rural areas. The students and their parents were requested to take the same survey questionnaire respectively (student questionnaire and parent questionnaire) again after 12 months, which has been used in previous studies [17, 25]. The investigation took place between October and November. Details of the study protocol were described in a previous study [8]. This study was approved by the Ethics Review Committee of Shanghai University of Sport in 2016. Because of the study nature with minimum risk, only verbal assent and consent were required for in the study.

### Study participants

A total of 1611 school-aged children and adolescents (aged 9–18 years) were invited in the baseline survey in 2016, and 14 students were excluded from the study because their answers were out of the normal range (1–5 scores) or omitted in the questionnaires in 2016 or 2017. Therefore, 1597 students were included in 2016 (T0) and followed after 12 months in 2017 (T1). Among them, boys and girls were 48.2% (*n* = 770) and 51.8% (*n* = 827), respectively (see Table 1); 725 (45.4%) of them attended the schools in urban area and 872 (54.6%) in rural area; 629 (39.4%) of them were adolescents (13 to 17 years old) and 968 (60.6%) children (9 to 12 years old).

**Table 1:** Descriptive statistical analysis of the numeric variables

Study variables	Year 2016Mean ± SD (95% CI)	Year 2017 Mean ± SD (95% CI)	*P* value
Age	11.90 (11.78,12.03)	12.90 (12.78,13.03)	
Gender (% boys)	48.22 (45.77,50.67)	48.22 (45.77,50.67)	
BMI (kg/m^2^)	18.46 (18.31,18.61)	19.16 (18.99,19.33)	< 0.001
MVPA days (0–7 days)	3.83 ± 1.95 (3.73, 3.92)	3.74 ± 1.93 (3.65, 3.84)	0.106
Attitude to PA (0–5 score)	3.73 ± 1.10 (3.68, 3.79)	3.98 ± 1.05 (3.93, 4.03)	< 0.001
Neighborhood sport events	2.37 ± 1.67 (2.31, 2.43)	2.70 ± 1.24 (2.64, 2.76)	< 0.001
Neighborhood exercise skill training	1.97 ± 1.09 (1.92, 2.03)	2.34 ± 1.26 (2.28, 2.40)	< 0.001
Neighborhood sport organization(%YES)	20.66 (18.75,22.72)	32.37 (30.12,34.71)	< 0.001
Neighborhood sport facility(%YES)	61.24 (58.82,63.60)	71.95 (69.69,74.10)	< 0.001
School exercise facility	4.11 ± 0.99 (4.06, 4.16)	4.20 ± 0.97 (4.15, 4.24)	0.013
School PE class	4.37 ± 0.88 (4.33, 4.41)	4.42 ± 0.82 (4.38, 4.46)	0.042
School teacher support	4.15 ± 1.07 (4.10, 4.20)	4.27 ± 0.99 (4.22, 4.31)	0.001
School extra PA time	3.92 ± 1.14 (3.87, 3.98)	4.14 ± 1.04 (4.09, 4.19)	< 0.001
School PA environment	3.94 ± 1.08 (3.89, 3.99)	4.16 ± 1.02 (4.11, 4.21)	< 0.001
Friend support	3.92 ± 1.18 (3.87, 3.98)	4.13 ± 1.05 (4.08, 4.18)	< 0.001
Friend accompany	3.96 ± 1.18 (3.91, 4.02)	4.14 ± 1.07 (4.09, 4.19)	< 0.001
Parent PA days (0–7 days)	3.06 ± 2.15 (2.95, 3.16)	3.15 ± 2.15 (3.01, 3.22)	0.227

*MVPA* moderate and vigorous physical activity

*PA* physical activity

*CI* Confidence Interval

All neighborhood and school questions have a score range from 1 to 5

### Study procedure

Two trained research assistants were sent to the participant school for assisting the surveys conducted in 2016 and 2017. Before starting the initial survey, verbal assents from parents and teachers, and verbal consent from students were obtained. Detailed instructions for the survey were provided and all questions were answered. The survey questionnaires were completed in the classroom within ≤20 min, which included the perceived PA environment of the neighbourhood and school. Besides, the parents’ survey including weekly PA days was conducted off-campus by a parent questionnaire. The numeric identification code was assigned to the questionnaire. An experienced research assistant input data into a computer database, which was only accessed by authorized project staff.

### Study variables

#### PA levels

The modified Chinese-version of the International Physical Activity Questionnaire Short Form (IPAQ-SF) was used to assess the PA levels of the students, which has been used in previous studies [8, 16, 24]. Student’s responses to the question “How many days did you have moderate to vigorous physical activity (MVPA), i.e., increased breathing rates and felt sweating, more than 60 minutes in last 7 days” were categorized into three groups: the sedentary group with exercise 0 to 1 day, physically inactive group with exercise 2 to 4 days, and physically active group with exercise 5 to 7 days. Similarly, parent’s PA levels (provided by parent survey) also were categorized into three groups according to their response for MVPA days in the past week more than 30 min, i.e., sedentary, physically inactive, and physically active groups.

### Neighbourhood factors

Students were requested to answer 4 questions about the neighbourhood (Qn): Qn-1 “There were game/sport events held for children and/or adolescents in your neighbourhood during last year”(neighbourhood sport events) with possible answers scored from 1 - never, 2 - not often, 3 - so-so, 4 - often, or 5 - very often. Qn-2 “There were free sport and/or exercise skills/training for children and/or adolescents in your neighbourhood during last year” (neighbourhood exercise skill training) with the same 5-score answers as in Qn-1. Qn-3 “Are there sport organizations available for children and/or adolescents in your neighbourhood” (1 – yes or 2 – no) (neighbourhood sport organization). Qn-4 “Are there sport facilities for children and/or adolescents conveniently located in your neighbourhood” (1 – yes or 2 – no) (neighbourhood sport facility). The answers for Qn-1 and Qn-2 were grouped into three categories, i.e., groups with negative (answers 1 and 2), neutral (answer 3), and positive (answers 4 and 5) neighbourhood PA environment for simplicity.

### School factors

Questions for school PA environment (Qs) included Qs-1 “School exercise facilities and equipment can meet my needs for physical activity and exercise”(School exercise facility); Qs-2 “PE class plays an important role for me to participate in physical activity and exercise”(School PE class); Qs-3 “School teachers encourage me to participate in physical activity and exercise”(School teacher encourage); Qs-4 “School provides extra time for physical activity and exercise”(School extra PA time); and Qs-5 “School has a desirable culture/environment for physical activity and exercise”(School PA culture/environment). All these questions had five possible answers: 1 – completely disagree, 2 – disagree, 3 – not sure, 4 – agree, or 5 – completely agree. Also, there were two more questions about student’s friends: Qs-7 “Friends often encourage me to participate in physical activity and exercise training”(Friend encouragement) and Qs-8 “Friends often participate in physical activity and exercise training with me”(Friend accompany) with 5-score answers from completely disagree (score 1) to completely agree (score 5). These questions were also categorized into three groups: non-desirable/negative (combined answers 1 and 2), neutral (answer 3), and desirable/positive (combined answers 4 and 5) school PA environment groups, respectively.

### Attitude to PA/exercise

Student’s attitude to PA/exercise was assessed by the survey question “Your attitude to participating in physical activity and/or exercise in future” 1 – don’t like PA/exercise and won’t plan to participate; 2 – will start PA/exercise; 3 – will do more PA/exercise, but not every day; 4 – will try to do PA/exercise every day; or 5 – will keep exercise every day. The responses were grouped into three categories: negative attitude (answer 1); positive attitude (combined answers 2 and 3); and a very positive attitude (combined answers 4 and 5).

### Statistical analysis

Continuous variables were presented as means±standard deviations and categorical variables as percentages. Differences in numeric scores between T0 and T1 were examined using paired t-test. Furthermore, multiple logistic regression was used to examine the associations between factors and students with a physically active lifestyle (exercise ≥5 days during the last 7 days) at baseline and change or maintenance of MVPA level(i.e. positive, PA days in 2017 > 2016; negative MVPA days in 2017 < 2016 and maintenance MVPA days in 2017 = 2016) at 12-month follow-up. At first, a full model (supplementary material Tables 1 and 2) was established to screen potential factors associated with MVPA level, students’ attitude to PA, neighbourhood and school factors were included in the model, and any factors were at least some indication for an association with the outcome (i.e., *p* <  0.2) was subsequently included in the final model.

Relative risk (RR) and 95% confidence interval (CI) were estimated to quantify the difference based on Chi-square test or logistic regression analysis. *P*-value < 0.05 was taken to indicate statistical significance. All statistical analyses were performed using ﻿Stata software (Stata 15.0, Stata Corporation, College Station, TX).

## Results

Data for the current analysis were taken from the 2016 Physical Activity and Fitness in China—the Youth Study (PAFCTYS) project, and follow-up data after 12 months were analysed. Table 1 describes the basic sample characteristics and scores from the questionnaire. There were no significant differences in the number of days students engaged in MVPA between 2016 (3.8 ± 2.0 days) and 2017 (3.7 ± 2.0 days) (*p* = 0.106) or in their parents’ PA behaviour (*p* = 0.23). Overall, the students’ MVPA was relatively low; only 36.8% (2016) and 33.6% (2017) of students were physically active, and boys were more physically active than girls (RR 1.55, 95% CI 1.10, 2.20). Moreover, children may be more physically active than adolescents (RR 1.33,95% CI 0.93, 1.90). Furthermore, more sport organizations and sport facilities were available for students in 2017 than in 2016 (Table 1).

The cross-sectional results show the correlates of students’ MVPA levels in Table 2 (the results of the full model are presented in supplementary material Table 1). In 2016, in a relatively relaxing environment, more extracurricular factors contributed to MVPA, with students reporting being physically active 5 to 7 days per week. Students who answered “yes” to having sport facilities (RR 1.82, 95% CI 1.28, 2.58) and sport organizations (RR 2.60, 95% CI 1.42, 5.00) in the neighbourhood were respectively 82 and 160% more likely to be physically active. Moreover, students had a higher MVPA level if they lived in a community with more sport events (RR 2.16, 95% CI 1.17, 4.00) and if they had more active parents (RR 1.99, 95% CI 1.25, 3.16). Students in a good PA environment spent more time on MVPA than those in a negative school PA environment (RR 2.07, 95% CI 1.21, 3.54). Notably, students’ attitudes towards PA was the most significant factor associated with students’ MVPA levels (RR 4.64, 95% CI 1.46,14.76).

**Table 2:** Positive contributors predict students to be physically active at baseline

Variables		RR	95% CI		p
Children vs Adolescent		1.33	0.93	1.90	0.117
Gender Boys vs girls		1.55^b^	1.10	2.20	0.013
Attitude to PA Positive vs negative		4.61^b^	1.43	14.89	0.011
Neighborhood sport event positive vs. negative		2.15^b^	1.16	3.99	0.015
Neighborhood sport organization Yes vs. no		2.60^b^	1.38	4.87	0.003
Neighborhood sport facility Yes vs. no		1.75^b^	1.23	2.49	0.002
School PA culture/environment Positive vs negative		2.07^b^	1.21	3.54	0.008
Friends accompany Positive vs. negative		1.60^b^	1.00	2.55	0.048
Parents PA days	neutral vs negative	1.64^b^	1.10	2.44	0.015
	Positive vs negative	1.97^b^	1.23	3.13	0.005

*RR* relative risk

*CI* confidence interval

^b^: represent the *p* < 0.05

Table 3 summarizes the factors that predict maintaining and increasing students’ MVPA levels (the results of the full model are presented in supplementary material Table 2). The results demonstrated that girls (RR 1.38, 95% CI 1.10, 1.73) were more likely to improve their MVPA than boys, and students’ MVPA level was more likely to decrease instead of increase with age (RR 0.95, 95% CI 0.91, 0.99). Interestingly, none of the neighbourhood factors contributed to maintaining or promoting MVPA behaviour. Schools play an important role in maintaining and developing students’ PA behaviour. Extra PA time at school (RR 1.20, 95% CI 0.98, 1.47) was a determinant of maintaining students’ MVPA level but was not significant for improving the level of activity. Meanwhile, PE class at school may be a potential factor for students MVPA level maintenance (RR 1.25, 95% CI 0.96, 1.63) and development (RR 1.31, 95% CI 0.99, 1.72). These may indicate that students have no time playing out of school. Furthermore, physically active parents and friends have a positive influence for students’ MVPA level. Friends’ encouragement of PA behaviour is an important determinant of MVPA improvement (RR 1.35, 95% CI 1.11, 1.63), whereas parents’ PA behaviour was a helpful contributor to their children’s MVPA maintenance (RR 1.42, 95% CI 1.16, 1.75). Similar to the results of the cross-sectional model, students’ attitudes towards PA affected not only the level of MVPA but also its maintenance (RR 1.37, 95% CI 1.02,1.84) and development (RR 1.31, 95% CI 1.04,1.64).

**Table 3:** Factors determining maintain and increase in MVPA days from 2016 to 2017

	Variables	RR	95% CI		p
Negative	Reference				
Stable	Age	1.02	0.97	1.08	0.407
	Gender Girls vs boys	0.87	0.66	1.15	0.339
	Attitude to PA	1.36^b^	1.01	1.83	0.040
	Neighborhood sport facility yes vs no	1.01	0.73	1.40	0.940
	School PE class	1.31	0.91	1.89	0.141
	School extra PA time	1.25^a^	0.96	1.63	0.092
	Friends encourage	1.13	0.89	1.44	0.250
	Parents PA behavior	1.42^b^	1.15	1.74	0.001
Positive	Age	0.95^b^	0.91	0.99	0.024
	Gender Girls vs boys	1.38^b^	1.10	1.73	0.005
	Attitude to PA	1.31^b^	1.04	1.64	0.022
	Neighborhood sport facility yes vs no	1.14	0.89	1.47	0.308
	School PE class	1.31^a^	0.99	1.72	0.055
	School extra PA time	1.20^a^	0.98	1.47	0.077
	Friends encourage	1.35^b^	1.11	1.63	0.003
	Parents PA behavior	1.13	0.96	1.33	0.150

Negative: students’ MVPA days in 2017 < 2016

Stable: students’ MVPA days in 2017 = 2016

Positive: students’ MVPA days in 2017 > 2016

*RR* relative risk

*CI* confidence interval

^a^: represent the *p* < 0.1; ^b^: represent the *p* < 0.05

## Discussion

In the current study, we explored the factors associated with students’ MVPA level at baseline and the change in the number of days of MVPA with a 12-month follow-up. Our results revealed no significant difference in MVPA level after a 12-month follow-up. Both school factors and neighbourhood factors contributed to students’ MVPA level. In the longitudinal model, students’ attitudes towards PA were found to affect both MVPA maintenance and improvement, while PE class were also found to be a potential factor with a similar effect. Furthermore, extra PA time and MVPA behaviour of students’ parents contribute to maintaining but not significantly increasing the MVPA level. However, students with more encouragement from friends tended to increase their number of days of MVPA in the 12-month follow-up.

In the present study, there were no significant differences in the number of days of MVPA and levels between 2016 and 2017, although some students changed their PA behaviour positively or negatively. However, previous studies identified that MVPA decreased with age in children and adolescents [26–28]. Two large-scale questionnaire studies have shown that the proportion of students who met the MVPA guidelines ranged from 29.9% in 2016 to 34% in 2017 in China [9, 10]. This indicates that some factors influence the changing trend. The integral trend might be partially influenced by “Healthy China 2030”, which was issued in October 2016 and urged students to participate in MVPA for more than 60 min/day and pursuing a goal to have more than 25% of students achieve an excellent fitness rating.

The results demonstrated that boys were more physically active than girls, which is similar to that found in previous studies [29, 30]. However, the attitude towards PA, the most significant contributor to MVPA level in our study, but did not differ between girls and boys (not shown in the results), indicating that the sex differences in MVPA level resulted from other factors. However, attitude towards PA was a powerful predictor of MVPA level, as identified in a previous study showing that students who think PA is good and engage in enjoyable PA spend more time in MVPA at school [31]. Neighbourhood PA facility was also a significant contributor to students’ MVPA level. This result is consistent with other studies showing that available PA facilities are positively associated with MVPA [18, 32]. In addition, students who live in a neighbourhood with a sport organization and events are more likely to be physically active, as such neighbourhoods provide more PA opportunities for children and adolescents. A study from the UK suggested that neighbourhood-based PA is critical for helping students to increase MVPA but not for sedentary behaviour reduction [16]. All of the above neighbourhood factors might help to remove barriers to students’ participation in PA, but more intervention studies are needed to explore the mechanism. Moreover, active students have active parents, and the modelling effect of parents on their children’s PA behaviour has been identified [33]. There is a positive relationship between neighbourhood PA opportunity availability and parents’ number of days of MVPA (not shown in the results). Perhaps there is a mutually promoting relationship, but this is not within the scope of this study. Clearly, both the neighbourhood PA environment and parents’ PA behaviour are significantly associated with students’ MVPA level. It will be useful for increasing students’ MVPA outside of school that improving neighbourhood environment of activity.

For the school factors, the results demonstrated that the school PA culture/environment was significantly associated with students’ physical activity, but school PA facilities and PE classes were not. A possible reason is that these two factors were similar among different schools, with few specific differences in the number of PE classes and the PA infrastructure in Chinese school, such as basketball and football courts. Moreover, a systematic review found that facilities and equipment are considered important for physical activity promotion; if wider school policies do not encourage and support the use of these by all students, the overall impact on physical activity is likely to be negligible [13]. Therefore, a good school PA culture/environment may be more important than the physical environment [17]. However, we did not find the significant association between friend factors and MVPA level, whether an encouraging or accompanying role. This finding differs from previous studies reporting that friends’ encouragement and engagement were positively associated with MVPA [21], the reason may be that relationship between friends’ support and MVPA was mediated by self-efficacy and enjoyment [34]. In China, infrastructural exercise facilities are similar among schools, but neighbourhood environments are not, which might cause differences in MVPA levels. Our results partially identified this condition. Overall, the combination and interaction of school and neighbourhood factors appear to influence adolescent PA, rather than being influenced by a single characteristic of the school or neighbourhood. .

In the longitudinal model, we found that students’ number of days of MVPA were more likely to decrease their MVPA level with age. The result is consistent with a meta-analysis of cohort studies [22], and it is also a global issue that causes many health problems. Notably, students’ attitudes towards PA not only related to a higher MVPA level but also indicated a positive change in MVPA behaviour, and these results were supported by previous studies [35–37]. This can also be explained by autonomous motivation, a component of self-determination theory [38], which positively predicts PA participation [39, 40]. Furthermore, higher PE motivation was positively associated with PA and sport participation [41], which can be explained by the trans-contextual model of motivation; this is also why PE class and extra PA time were potential factors for maintaining and promoting MVPA levels [42]. More health- and exercise-related knowledge were provided in PE class may improve the awareness and attitude of student to PA, which will increase the PA and physical fitness level [43, 44]. Interestingly, we found that girls were more likely to increase their MVPA level at the 12-month follow-up than boys, which is consistent with a previous systematic review that found that non-organized PA declined among adolescent boys but not girls [45]. Moreover, based on the relatively lower MVPA level among girls at baseline, they have a higher probability of increasing their MVPA level. Finally, students with physically active parents tended to maintain stable MVPA levels and tended to increase their MVPA levels when they had more support from friends. Both cross-sectional and longitudinal studies have identified the positive effect of parents and friends on students’ PA behaviour [46, 47], but these effects may occur at different stages. Parental modelling of PA plays an integral role in establishing social norms regarding activity before puberty [48], and the emergent influence of peers in modelling behaviour may replace the influence of parental modelling gradually as the child matures [33]. A recent study reported that students with high PA levels participated more in organized sports [49], and another study suggested that organized and team sports may reinforce social norms and conformity to peer behaviour [50]. Accordingly, more organized sports are held in neighbourhoods, and schools may engage students’ parents and friends to also join activities, which will help to increase students’ MVPA levels. Integrating parent and peer support into intervention programmes may also be an effective strategy for improving PA behaviour [51]. In addition, the results of a feasibility cluster randomized controlled trial of a peer-led school-based intervention to increase the PA of adolescent girls demonstrated that peer-supporter training for supporting friends in participating in PA was effective for stemming the age-related decline in PA in adolescent girls’ PA [52]. More similar studies need to be conducted to explore an effective and cost-effective way to increase students’ MVPA levels through peer or parent support.

Our study has some obvious weaknesses. First, we assessed the MVPA level with self-report data, which causes bias in MVPA assessment. With this method, we cannot distinguish between in-school and after-school MVPA. Moreover, we used the number of days of students participating in MVPA for more than 60 min, as many students cannot estimate the precise duration of MVPA behaviour, and they cannot clearly distinguish among low-intensity, moderate-intensity, and vigorous-intensity physical activity. Therefore, many students did not answer this question. This may account for some of the lower precision of the information. In the future, more studies are needed to develop an accurate and efficient tool to estimate PA time for students, especially for younger students, in large-scale investigations. Second, we measured the school and neighbourhood PA environment via self-perception instead of objective assessment instruments, which may limit the implementation of improvements in the environment. Future studies should apply an objective assessment instrument to examine the association between these influential factors and MVPA in detail. Consequently, we would be able to better understand the association between different influential factors and the distribution of the area and time of PA behaviour. In addition, further studies need to explore the transformation of factors influencing PA behaviour with age, which will provide more precise information to support interventions for PA behaviour.

The findings of our study provide epidemiological evidence for the need for MVPA interventions in children and adolescents in the future. This evidence is also important for developing policies for promoting school-aged children’s participation in MVPA. In our results, improving the neighbourhood PA environment and attitudes cannot translate into MVPA level evaluation, indicating that there are some influential factors impacting the MVPA of school-aged children significantly with increasing age and school grade. A study from China reviewed the role of policy in preventing fitness decreases and revealed that the policy alone did not seem effective [53]. That is to say, a single health policy alone cannot improve the MVPA level of school-aged children. In China, studying is the top priority for school-aged children, and thus, most of their time is used in studying rather than engaging in PA. In the future, concurrent education and health policy may be effective for increasing the MVPA level of school-aged children. Furthermore, a change in behaviour is not an isolated problem, as it is influenced by many factors and at a distinct level [54, 55]. Future studies should explore influential factors more comprehensively, and interventions should be full-scale and multilevel.

## Conclusion

In conclusion, both neighbourhood and school factors affect students’ MVPA, but school plays a more meaningful role in maintaining and promoting students’ MVPA levels. In addition, individual attitudes towards PA may be more important than the external environment for student MVPA levels. More attention should be given to older boys, as they are more likely to decrease their MVPA. Our research demonstrates that students’ attitudes towards PA and school factors should be targets for future intervention programmes to promote MVPA; future PA promotion programmes should consider the social-ecological model [34], as well as individual, school, neighbourhood and other factors. Education aimed at improving individual attitudes towards PA might be the most cost-effective method to increase students’ MVPA levels. More studies with large samples and objective assessments should be conducted to explore the determinants of MVPA.

## Supplementary Information

**Additional file 1: Supplementary material Table 1.** Multiple logistic regression for the predictors of students MVPA level at baseline. This table is the full model of the cross-sectional multiple logistic regression, which was used to screen the potential factors associated with students’ MVPA level. Gender, Age category, Attitude to PA, Neighbourhood sport event, Neighbourhood sport organization, Neighbourhood sport organization, Neighbourhood sport facility, School PA culture/environment, Friends accompany and parents PA days had been included into the final model. **Supplementary material Table 2.** Multiple logistic regression for the factors that affect the change of students MVPA behaviour. This table is the full model of the longitudinal multiple logistic regression, which was used to screen the potential factors could predict the students’ MVPA level. Age, Gender, Attitude to PA, Neighbourhood sport facility, School PE class, School extra PA time, Friends encourage and parents PA days had been included into the final model.

## Data Availability

The datasets used and/or analysed during the current study are available from the corresponding author on reasonable request.

## Abbreviations

PA: Physical activity
MVPA: Moderate and vigorous physical activity
SED: Sedentary behaviour
BMI: Body mass index
RR: Relative-risk
CL: Confidence interval
Qn: Question about neighbourhood
Qs: Questions for school PA environment
ANOVA: Analysis of variance
PAFCTYS: Physical activity and fitness in China-The Youth Study
