# Supplementary material for: Determinants of student’s physical activity: a 12-month follow-up study in Ningxia province
Source: BMC Public Health. 2021 Mar 16;21:512. doi: 10.1186/s12889-021-10525-1 (PMC7968158; doi:10.1186/s12889-021-10525-1)
Supplement: Supplementary file 1 — Additional file 1: Supplementary material Table 1. Multiple logistic regression for the predictors of students MVPA level at baseline. This table is the full model of the cross-sectional multiple logistic regression, which was used to screen the potential factors associated with students’ MVPA level. Gender, Age category, Attitude to PA, Neighbourhood sport event, Neighbourhood sport organization, Neighbourhood sport organization, Neighbourhood sport facility, School PA culture/environment, Friends accompany and parents PA days had been included into the final model. Supplementary material Table 2. Multiple logistic regression for the factors that affect the change of students MVPA behaviour. This table is the full model of the longitudinal multiple logistic regression, which was used to screen the potential factors could predict the students’ MVPA level. Age, Gender, Attitude to PA, Neighbourhood sport facility, School PE class, School extra PA time, Friends encourage and parents PA days had been included into the final model. [file 12889_2021_10525_MOESM1_ESM.docx]

Supplementary material

| Variables | | RR | p | 95% CI | |
| --- | --- | --- | --- | --- | --- |
| Children vs Adolescent | | 1.33 | 0.1232 | 0.93 | 1.92 |
| Gender boys vs girls | | 1.52 | 0.0193 | 1.08 | 2.15 |
| BMI category | Overweight vs normal | 1.20 | 0.5406 | 0.67 | 2.16 |
|  | Obesity vs normal | 0.92 | 0.8080 | 0.46 | 1.84 |
| Student attitude to PA | Neutral vs negative | 1.44 | 0.5488 | 0.44 | 4.72 |
|  | Positive vs negative | 4.41 | 0.0156 | 1.32 | 14.70 |
| Neighborhood sports event | Neutral vs negative | 1.48 | 0.0901 | 0.94 | 2.31 |
|  | Positive vs negative | 2.17 | 0.0248 | 1.10 | 4.28 |
| Neighborhood exercise skill training | Neutral vs negative | 1.10 | 0.7070 | 0.66 | 1.83 |
|  | Positive vs negative | 0.93 | 0.8567 | 0.41 | 2.10 |
| Neighborhood sports organization | No vs yes | 0.37 | 0.0027 | 0.20 | 0.71 |
| Neighborhood sports facility | No vs yes | 0.57 | 0.0022 | 0.40 | 0.82 |
| School PA facility | Neutral vs negative | 0.62 | 0.2173 | 0.29 | 1.32 |
|  | Positive vs negative | 1.08 | 0.8139 | 0.58 | 2.00 |
| School PE class | Neutral vs negative | 0.81 | 0.6482 | 0.32 | 2.02 |
|  | Positive vs negative | 0.75 | 0.4756 | 0.34 | 1.64 |
| School extra PA time | Neutral vs negative | 1.16 | 0.6555 | 0.60 | 2.25 |
|  | Positive vs negative | 1.06 | 0.8498 | 0.60 | 1.84 |
| School PA culture/environment | Neutral vs negative | 1.10 | 0.7687 | 0.58 | 2.11 |
|  | Positive vs negative | 2.03 | 0.0206 | 1.12 | 3.71 |
| School teachers encourage | Neutral vs negative | 1.18 | 0.6568 | 0.57 | 2.44 |
|  | Positive vs negative | 1.62 | 0.1360 | 0.86 | 3.03 |
| Friends accompany | Neutral vs negative | 0.62 | 0.1494 | 0.33 | 1.18 |
|  | Positive vs negative | 1.51 | 0.1180 | 0.90 | 2.55 |
| Friends encourage | Neutral vs negative | 1.48 | 0.2432 | 0.77 | 2.84 |
|  | Positive vs negative | 1.11 | 0.7030 | 0.65 | 1.91 |
| Parents PA behavior | Neutral vs negative | 1.63 | 0.0178 | 1.09 | 2.43 |
|  | Positive vs negative | 1.93 | 0.0063 | 1.20 | 3.10 |

Table 1.Multiple logistic regression for the predictors of students MVPA level at baseline

RR: relative risk ; CI: confidence interval

Table 2. Multiple logistic regression for the factors that affect the change of students MVPA behavior.

| Variables | | RR | p | 95% CI | |
| --- | --- | --- | --- | --- | --- |
| Adolescent/Children | Stable vs negative | 1.03 | 0.2795 | 0.97 | 1.09 |
|  | Positive vs negative | 0.96 | 0.0600 | 0.91 | 1.00 |
| Gender boys vs girls | Stable vs negative | 0.86 | 0.2929 | 0.65 | 1.14 |
|  | Positive vs negative | 0.72 | 0.0051 | 0.58 | 0.91 |
| BMI category | Stable vs negative | 1.02 | 0.8942 | 0.81 | 1.28 |
|  | Positive vs negative | 0.99 | 0.9036 | 0.83 | 1.19 |
| Student attitude to PA | Stable vs negative | 1.32 | 0.0729 | 0.97 | 1.78 |
|  | Positive vs negative | 1.27 | 0.0439 | 1.01 | 1.61 |
| Neighborhood sports event | Stable vs negative | 1.10 | 0.4360 | 0.87 | 1.39 |
|  | Positive vs negative | 1.19 | 0.0676 | 0.99 | 1.43 |
| Neighborhood exercise skill training | Stable vs negative | 0.96 | 0.7553 | 0.76 | 1.22 |
|  | Positive vs negative | 0.91 | 0.3167 | 0.76 | 1.09 |
| Neighborhood sports organization  No vs yes | Stable vs negative | 1.05 | 0.7781 | 0.74 | 1.49 |
|  | Positive vs negative | 1.07 | 0.6268 | 0.81 | 1.41 |
| Neighborhood sports facility  No vs yes | Stable vs negative | 1.06 | 0.7329 | 0.75 | 1.50 |
|  | Positive vs negative | 1.20 | 0.1898 | 0.92 | 1.56 |
| School PA facility | Stable vs negative | 1.20 | 0.5994 | 0.61 | 2.36 |
|  | Positive vs negative | 0.95 | 0.8443 | 0.58 | 1.55 |
| School PE class | Stable vs negative | 1.33 | 0.1393 | 0.91 | 1.93 |
|  | Positive vs negative | 1.28 | 0.0949 | 0.96 | 1.70 |
| School extra PA time | Stable vs negative | 1.27 | 0.1162 | 0.94 | 1.70 |
|  | Positive vs negative | 1.19 | 0.1408 | 0.94 | 1.49 |
| School PA culture/environment | Stable vs negative | 1.04 | 0.8058 | 0.76 | 1.41 |
|  | Positive vs negative | 0.93 | 0.5756 | 0.74 | 1.18 |
| School teachers encourage | Stable vs negative | 0.84 | 0.2365 | 0.63 | 1.12 |
|  | Positive vs negative | 1.12 | 0.3558 | 0.88 | 1.42 |
| Friends accompany | Stable vs negative | 1.13 | 0.4277 | 0.83 | 1.54 |
|  | Positive vs negative | 1.00 | 0.9881 | 0.79 | 1.26 |
| Friends encourage | Stable vs negative | 1.05 | 0.7349 | 0.78 | 1.42 |
|  | Positive vs negative | 1.32 | 0.0220 | 1.04 | 1.68 |
| Parents PA behavior | Stable vs negative | 1.41 | 0.0011 | 1.15 | 1.74 |
|  | Positive vs negative | 1.12 | 0.1891 | 0.95 | 1.31 |

RR: relative risk ; CI: confidence interval
